# Expression profile of *Caenorhabditis elegans* mutant for the Werner syndrome gene ortholog reveals the impact of vitamin C on development to increase life span

**DOI:** 10.1186/1471-2164-15-940

**Published:** 2014-10-27

**Authors:** Alexandra Dallaire, Sophie Proulx, Martin J Simard, Michel Lebel

**Affiliations:** Centre de Recherche sur le Cancer de l’Université Laval, Hôpital Hôtel-Dieu de Québec (CHU de Québec Research Center), 9 McMahon Sreet, Québec City, G1R 2 J6 Canada

## Abstract

**Background:**

Werner Syndrome (WS) is a rare disorder characterized by the premature onset of a number of age-related diseases. The gene responsible for WS encodes a DNA helicase/exonuclease protein believed to affect different aspects of transcription, replication, and DNA repair. *Caenorhabditis elegans (C. elegans)* with a nonfunctional *wrn-1* DNA helicase ortholog also exhibits a shorter life span, which can be rescued by vitamin C. In this study, we analyzed the impact of a mutation in the *wrn-1* gene and the dietary supplementation of vitamin C on the global mRNA expression of the whole *C. elegans* by the RNA-seq technology.

**Results:**

Vitamin C increased the mean life span of the *wrn-1*(*gk99*) mutant and the N2 wild type strains at 25°C. However, the alteration of gene expression by vitamin C is different between *wrn-1*(*gk99*) and wild type strains. We observed alteration in the expression of 1522 genes in *wrn-1*(*gk99*) worms compared to wild type animals. Such genes significantly affected the metabolism of lipid, cellular ketone, organic acid, and carboxylic acids. Vitamin C, in return, altered the expression of genes in *wrn-1*(*gk99*) worms involved in locomotion and anatomical structure development. Proteolysis was the only biological process significantly affected by vitamin C in wild type worms.

**Conclusions:**

Expression profiling of *wrn-1*(*gk99*) worms revealed a very different response to the addition of vitamin C compared to wild type worms. Finally, vitamin C extended the life span of *wrn-1*(*gk99*) animals by altering biological processes involved mainly in locomotion and anatomical structure development.

**Electronic supplementary material:**

The online version of this article (doi:10.1186/1471-2164-15-940) contains supplementary material, which is available to authorized users.

## Background

WS is a human autosomal recessive disorder characterized by genomic instability and the premature onset of a number of age-related diseases [1–4]. The defective enzyme responsible for WS possesses a 3′-5′ exonuclease activity in addition to a 3′-5′ helicase activity [5, 6] and is involved in DNA repair, replication, transcription, and telomere maintenance [7–11]. We previously generated a mouse model with a deletion in the helicase domain of the murine *WRN* homologue [12] that recapitulates most of the WS phenotypes, including an abnormal hyaluronic acid excretion, higher reactive oxygen species (ROS) levels, dyslipidemia, increased genomic instability, and cancer incidence [13, 14]. Overall, such mutant mice have a 10-15% decreased of their mean life span [15, 16]. Interestingly, the treatment of *Wrn* mutant mice with vitamin C reverted the life span of such animals to the normal wild type phenotype [16].

The WRN protein is a member of the RecQ family of DNA helicases [3]. It is highly conserved across species including in invertebrates such as the small worm *Caenorhabditis elegans (C. elegans).* Interestingly, the exonuclease and the DNA helicase domains homologous to the human WRN protein are encoded by two different genes in *C. elegans* [17]. The *C. elegans wrn-1* gene codes for the ATP-dependent 3′-5′ DNA helicase capable of unwinding a variety of DNA structures [18]. Notably, it has been shown that the RNAi knockdown of the *C. elegans wrn-1* gene shortens the life span, increases sensitivity to DNA damage, and accelerates aging phenotypes [17]. Similarly, a *C. elegans* strain lacking the expression of the *wrn-1* helicase protein (*wrn-1*(*gk99*)) also exhibit a shorter life span when grown at 25°C [19]. Interestingly, supplementation of vitamin C normalizes the median life span of the *wrn-1*(*gk99*) mutant worms [19] as seen in mice lacking part of the helicase domain of the Wrn protein [16]. Thus, the *wrn-1*(*gk99*) mutant worm model gives us the opportunity to examine the global mRNA expression of a whole animal upon vitamin C treatment.

In this study, we analyzed the global mRNA expression of wild type and *wrn-1*(*gk99*) mutant *C. elegans* in the presence or absence of vitamin C by RNA-seq. RNA-seq has been shown to be quantitatively accurate over a larger range of expression levels than previous methods, such as microarrays [20–24]. Our results indicate that *wrn-1* mutant animals showed significant changes in biological processes affecting several carbon structures (including lipid metabolisms) and oxidation-reduction reactions. Although supplementation of vitamin C significantly increased the life span of both wild type and *wrn-1* mutant worms, no gene was similarly affected by vitamin C in these strains. Vitamin C altered the expression of genes in *wrn-1*(*gk99*) worms involved in locomotion and anatomical structure development. Proteolysis was the only biological process significantly affected by vitamin C in wild type worms. These results indicate that the expression profiling of *wrn-1*(*gk99*) worms revealed a very different response to the addition of vitamin C compared to wild type worms.

## Results

### Vitamin C increases the life span of both *wrn-1*(*gk99*) and wild type *C. elegans*strains

**Figure 1:**
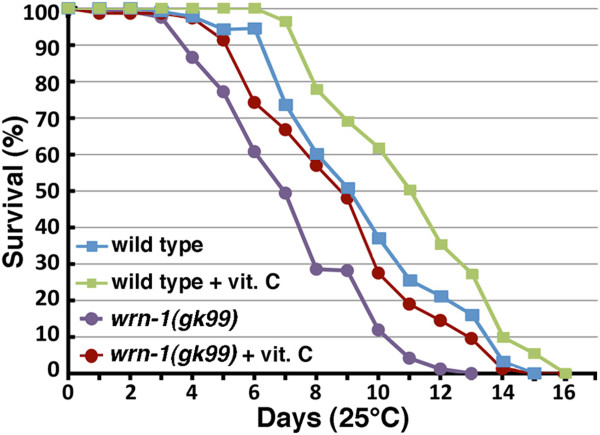
**Impact of vitamin C on the life span of wild type and** ***wrn- 1*** **mutant** ***C. elegans*** **strains.** The difference in longevity between vitamin C treated *wrn-1(gk99)* and untreated *wrn-1(gk99) C. elegans* strain grown at 25°C is significant (log rank test; *P* =1.83 × 10^-6^). The difference in longevity between vitamin C treated wild type (N2) and untreated wild type (N2) *C. elegans* strain grown at 25°C is significant (log rank test; *P* =2.07 × 10^-5^). All experiments were performed four to six times with 20 to 30 worms per genotype. *P*-values were obtained using the log-rank test method.

To assess the impact of vitamin C on longevity, we measured the life span of *wrn-1*(*gk99*) and wild type *C. elegans* strains grown at 25°C in the absence or presence of 10 mM ascorbate (vitamin C) as described before [19, 25]. As shown in Figure 1, the median life span of vitamin C treated *wrn-1*(*gk99*) worms was significantly increased by 26% (7.0 days *versus* 8.8 days) compared to untreated *wrn-1*(*gk99*) animals (log-rank test: *P* =1.83 × 10^-6^). These results replicate previous data obtained with these strains [19]. Interestingly, the median life span of vitamin C treated *wrn-1*(*gk99*) worms was close to the median life span of wild type worms (Figure 1). The median life span of vitamin C treated wild type worms was significantly increased by 22% (9.0 days *versus* 11.0 days) compared to the untreated wild type animals (log-rank test: *P* =2.07 × 10^-5^). These results indicate that vitamin C increased the life span of both strains by more than 20% at 25°C.

### Transcriptome characterization of the *wrn-1*(*gk99*) animals compared to the wild type strain

**Figure 2:**
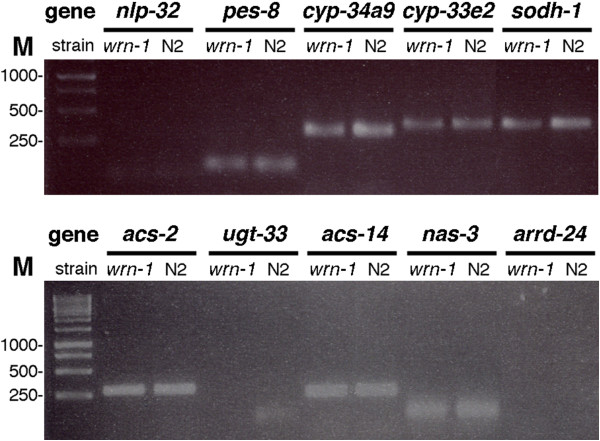
**Examples of RT-PCR products on 2% agarose gel.** Results are shown for *nlp-32*, *pes-8*, *cyp34a9*, *cyp-33e2*, *sodh-1*, *acs-2*, *ugt-33*, *acs-14, nas-3*, and *arrd-24* genes after 40 cycles of PCR. M = marker in base pairs; N2 = wild type strain; *wrn-1* = *wrn-1(gk99) C. elegans* strain.

To gain insight into the rescuing effect of vitamin C on the life span of *wrn-1*(*gk99*) worms, we first established the global expression profile of untreated wild type and mutant worms at the L4 stage by RNA-seq. By carefully monitored physiological developmental cues (such as vulva and gonad development), we noticed a three hours developmental delay in *wrn-1(gk99)* mutants compared to wild type animals. Therefore, mRNA was extracted based on the biological age (i.e. the exact developmental stage) and not on the chronological age of the animals for the RNA-seq analyses. Table 1 contains the number of raw reads for each biological replicate (including the vitamin C-treated worms). The normalized counts for each biological replicate are shown in the Additional file 1: Table S1 for all the *C. elegans* genes. A preliminary list of differentially expressed genes was generated by keeping the genes that showed a two-fold difference or more between wild type N2 and *wrn-1*(*gk99*) worms with an adjusted *P*-value <0.01. This first list contained 2528 genes (data not shown). We randomly chose 16 genes representing a range of base mean number from 4 to 3500 reads in both or in either N2 or *wrn-1* mutant animals based on the RNA-seq normalized data (list of genes in Table 2) to determine the minimum of reads required for a gene to be detected by RT-PCR in our analyses. We thus designed 16 pairs of primers to validate the differential expression of these genes by quantitative RT-PCR. The tubulin encoding gene (*tba-1*) was included as a control (no difference in *tba-1* expression between both strains). PolyA + RNA was thus extracted from three plates of wild type and from three plates of *wrn-1*(*gk99*) worms (each plate representing one biological replicate of worms at the L4 stage). The RT-PCR results are shown in Table 2 and the sequences of the primers are provided in the Additional file 2: Table S2. Every RT-PCR product was examined on a 2% agarose gel at the end of each run. Figure 2 shows examples of such gels for ten of these genes. A PCR product was detected only in samples that showed more than 145 reads by RNA-seq (Additional file 1: Table S1). For example, no band corresponding to *nlp-32* mRNA could be detected with the mRNA samples and no Ct could be detected by quantitative RT-PCR. The mean number of reads is below 134 for *nlp-32* mRNA in both the wild type and the *wrn-1*(*gk99*) worms (Table 2). Similarly, no *arrd-24* mRNA could be detected from wild type or *wrn-1*(*gk99*) worms mRNA pools even after 40 cycles of PCR (Figure 2). The normalized counts for the *arrd-24* gene were below 32 in both genotypes for all biological replicates (Table 2). No Ct could be detected by quantitative RT-PCR. In contrast, a band for the *ugt-33* gene was detected with mRNA from the wild type worms only (no band was detected for the *wrn-1*(*gk99*) worms) (Figure 2). The mean number of reads was lower than 145 for *ugt-33* in the *wrn-1*(*gk99*) worms but higher than 145 in the wild type worms (Table 2). Finally, Table 2 indicated expression differences for *pes-8*, *cyp-34a9*, *cyp-33e2*, *sodh-1*, *ugt-33*, *ech-9*, *nas-3*, *gpx-3*, *acs-2*, *acs-14*, *asah-1*, *lys-8*, Y46G5.20, and *daf-9* genes between the wild type and the *wrn-1*(*gk99*) strains by RT-PCR analyses and such results confirmed the RNA-seq data. A mean number of reads greater than 145 was found in at least one of the genotypes following the RNA-seq analysis for these genes (Table 2). Based on such results, we generated a final list of genes showing at least a two-fold difference in expression between wild type and mutant worms, an adjusted *P*-value <0.01, and a mean number of reads >145 in at least one of the worm strain. These criteria were applied to each comparison hereafter in this report. Additional file 3: Table S3 gives a final list of 1522 genes differentially expressed between wild type and *wrn-1*(*gk99*) worms. There were 907 down regulated and 615 up regulated genes respectively (by at least two-fold) in the *wrn-1*(*gk99*) worms compared to the wild type worms.

**Table 1:** ***C. elegans*** **genotypes used in this study**

Sample name	Number of reads
N2-1	18,288,816
N2-2	18,694,683
N2-3	23,406,553
N2-1 + vitC	19,647,258
N2-2 + vitC	19,580,669
N2-3 + vitC	24,862,436
wrn-1-1	37,999,237
wrn-1-2	26,388,834
wrn-1-3	21,700,537
wrn-1-1 + vitC	29,673,436
wrn-1-2 + vitC	15,031,408
wrn-1-3 + vitC	25,050,297

vitC =10 mM of vitamin C (ascorbate) in media.

**Table 2:** **Quantitative RT-PCR compared to RNA-seq data for 16 genes** ^**a**^

RNA-seq				RT-PCR		
Gene	Base mean in N2	Base mean in ***wrn-1***	Fold difference	ΔCt	Fold difference	P-value
*sodh-1*	465	5021	+10.79	3.02	+8.09	<0.001
*arrd-24*	4	32	+7.80	none	N/A	N/A
*gpx-3*	24	146	+6.21	1.07	+3.24	0.0012
*nas-3*	49	220	+4.51	1.78	+3.43	<0.001
*daf-9*	168	605	+3.60	1.48	+2.78	<0.001
*asah-1*	393	1200	+3.06	0.72	+1.65	0.0008
*acs-2*	594	1277	+3.30	1.80	+3.48	<0.001
*acs-14*	763	2289	+3.00	0.52	+1.43	0.0307
*ech-9*	155	449	+2.89	1.94	+3.84	0.0006
Y46G5.20	1182	381	-3.10	-2.05	-4.14	0.0001
*cyp-33e2*	1708	548	-3.12	-2.70	-6.52	<0.001
*cyp-34a9*	987	289	-3.41	-2.05	-4.15	<0.001
*pes-8*	256	66	-3.84	-2.06	-4.16	0.0204
*ugt-33*	269	69	-3.90	-1.67	-3.18	0.0008
*lys-8*	3405	155	-21.93	-4.45	-21.87	<0.001
*nlp-32*	135	5	-28.69	none	N/A	N\A
*tba-1* ^b^	12597	10618	-1.19	-0.12	-1.09	0.7083

^a^RT-PCRs were performed in triplicate and all values represent the means.

^b^The *tba-1* gene was used as a control (no difference of expression between N2 and *wrn-1* mutants).

The DAVID classification tool was used to classify differentially expressed genes by their functions in specific biological processes. Categorization of the genes in our list (Additional file 3: Table S3) revealed ten significantly up regulated biological processes (with a Benjamini *P*-value <0.05) in the *wrn-1*(*gk99*) worms compared to wild type worms (Table 3). Major biological processes that were up regulated in *wrn-1*(*gk99*) worms included oxidation-reduction processes, lipid, organic acid, cellular ketone, and carbohydrate metabolic processes (Table 3). There was no significant down regulated biological processes (with a Benjamini *P*-value <0.05).

**Table 3:** **Biological processes altered in** ***wrn-1(gk99) C. elegans*** **strain compared to the wild type N2 strain**

Up in ***wrn-1(gk99) C. elegans***		
Category	Benjamini ***P***-value	Genes
Organic acid metabolic process	1.34E-04	R09B5.6, F08A8.3, F56H11.4, F08A8.1, C12C8.2, F08A8.2, F28F8.2, M02D8.4, W08D2.4, C48B4.1, C44E4.3, C05E4.9, T21F4.1, T09B4.8, F01G10.3, K07E1.1, Y73F4A.2, F21F8.4, D1025.2, W09B6.1, F59B8.2, ZC416.6, ZK1290.12, F13D12.4, K10H10.2, ZK829.4, R08E5.2, R11A5.4
Cellular ketone metabolic process	1.78E-04	R09B5.6, F08A8.3, F56H11.4, F08A8.1, C12C8.2, F08A8.2, F28F8.2, M02D8.4, W08D2.4, C48B4.1, C44E4.3, C05E4.9, T21F4.1, T09B4.8, F01G10.3, K07E1.1, Y73F4A.2, F21F8.4, D1025.2, W09B6.1, F59B8.2, ZC416.6, ZK1290.12, F13D12.4, K10H10.2, ZK829.4, R08E5.2, R11A5.4
Lipid metabolic process	2.52E-04	R09B5.6, F08A8.3, F08A8.1, F56H11.4, F28F8.2, F08A8.2, K11D2.2, W08D2.4, Y52B11A.8, F09C8.1, C48B4.1, ZK6.7, T19H12.9, T05E7.1, F59F4.4, F46G10.4, F01G10.3, C40H1.7, H23N18.1, Y37E11AR.5, F11E6.5, T12B3.3, Y54G2A.45, F22E10.5, F21F8.4, F35C11.5, T19H12.10, W09B6.1, F28H7.3, ZC416.6, Y49E10.18, C31H5.6, C33C12.3, F54D11.1, W04C9.1
Oxidation-reduction	0.0072	T10B9.7, Y39A1A.19, T13C5.1, F08A8.2, C47A10.5, T08B1.3, F54D8.3, C48B4.1, K08C7.5, R02D3.1, C26F1.2, C02C2.1, F55B11.1, C11E4.2, R07B7.5, K06A4.5, F08F3.7, F36A2.3, R11D1.11, R04B5.5, F18E3.7, F59B8.2, ZC443.1, F13D12.4, ZK829.4, K12G11.3, K09D9.2, C30G12.2, Y5H2B.5, F01F1.6, C49G7.8
Carbohydrate metabolic process	0.0147	R09B5.6, C05E4.9, Y22F5A.4, Y22F5A.5, T19H12.9, F07A11.2, F07A11.5, R11F4.1, C02A12.4, H23N18.1, Y37E11AR.5, T12B3.3, Y73F4A.2, ZK678.8, F01F1.12, R11D1.11, R05F9.6, T19H12.10, D2096.3, H22K11.2, H02I12.1, C05C8.7, R09D1.10, Y46G5A.31, C33C12.3, R11A5.4

### Transcriptome characterization of *wrn-1*(*gk99*) and wild type strains treated with vitamin C

We compared the global mRNA expression of synchronized untreated worms with synchronized worms grown with vitamin C until the L4 stage. Lists of genes differentially expressed were generated by requiring that the absolute value of the fold change be higher than two, that the mean number of reads for each gene be higher than 145 for at least one of the genotype, and the adjusted *P*-value be lower than 0.01. When we compared the global expression of untreated *wrn-1*(*gk99*) worms with vitamin C treated *wrn-1*(*gk99*) worms, we found 311 genes to be differentially expressed (139 down and 172 up regulated genes, respectively) between both populations (Additional file 4: Table S4). DAVID analysis on this list of genes revealed enrichments for two main down regulated biological processes (Table 4). The main affected processes included genes involved in locomotion and anatomical structure development. No significant biological processes were up regulated (with a Benjamini P-value <0.05). When we compared the global expression of untreated wild type worms with vitamin C treated wild type worms, we found 296 genes to be differentially expressed (193 down and 103 up regulated genes, respectively) between both populations (Additional file 5: Table S5). DAVID analysis on this list of genes revealed a significant down regulation of only one set of genes involved in proteolysis (Table 4). Finally, the list of genes in the vitamin C treated wild type *versus* untreated wild type worms was very different from the list of differentially expressed genes in untreated *wrn-1*(*gk99*) *versus* vitamin C treated *wrn-1*(*gk99*) worms. Only four genes were regulated in a similar way in both lists and they included *mup-4*, C27D9.2 (down regulated), F12A10.1, and *ugt-18* (up regulated). The *mup-4* gene encodes a transmembrane protein required for junctional attachments between hypodermis and muscle [26]. The *ugt-18* gene encodes a protein with putative UDP- glucuronosyltransferase activities (referenced in http://www.ncbi.nlm.nih.gov/gene/179759). The C27D9.2 and F12A10.1 genes code for uncharacterized proteins (http://www.wormbase.org). These results indicate that although vitamin C increased the life span of both mutant and wild type worms, it affected different sets of genes and biological processes in both strains.

**Table 4:** **Comparison of biological processes altered in** ***wrn-1*** **mutant or wild type** ***C. elegans*** **strains treated with vitamin C**

Down in ***wrn-1***mutant + vitamin C ***vs***untreated ***wrn-1***mutant ***C. elegans***strain		
Category	Benjamini ***P***-value	Genes
Locomotion	0.0081	K07D8.1, C29F4.1, W05B2.1, T10E10.2, T28C6.6, T10E10.6, F46B3.5, F38A3.2, F55C10.3, T01B10.2, T28C6.4, C44C10.1, T22A3.8, F13H6.1, C26C6.3, F30B5.1, ZK270.1, Y4C6B.2, ZK678.5, W05B2.6, T07H6.3, K07C10.1, W05B2.5, Y41E3.2
Anatomical structure development	0.0065	C29F4.1, T10E10.2, T10E10.6, C29E4.1, C09G5.4, F38A3.2, T01B10.2, C44C10.1, F37B4.2, T07H6.3, F52B11.4, W05B2.1, T22B3.1, F27C1.8, T28C6.6, F55C10.3, T28C6.4, T22A3.8, F30B5.1, F11G11.10, B0491.2, F11G11.12, ZK270.1, W05B2.6, Y41E3.2, W05B2.5
**Up in** ***wrn-1*** **mutant + vitamin C** ***vs*** **untreated** ***wrn-1*** **mutant** ***C. elegans*** **strain**		
No significant category		
**Down in N2 + vitamin C** ***vs*** **untreated N2** ***C. elegans*** **strain**		
**Category**	**Benjamini** ***P*** **-value**	**Genes**
Proteolysis	0.0408	T23F4.4, ZK1037.10, F53A9.2, K07B1.1, W07B8.4, T19D2.1, W01A8.6, C29F3.2, B0222.4, F47H4.10, F42A10.8, Y95B8A.1, R10H1.5, C07D10.4
**Up in N2 + vitamin C** ***vs*** **untreated N2** ***C. elegans*** **strain**		
No significant category		

The life span of *wrn-1*(*gk99*) worms treated with vitamin C is similar to the life span of untreated wild type worms (Figure 1). We therefore compared the global expression of vitamin C treated *wrn-1*(*gk99*) worms with the global expression of untreated wild type worms. There were 375 down regulated and 181 up regulated genes in the vitamin C treated *wrn-1*(*gk99*) worms compared to untreated wild type worms, respectively (Additional file 6: Table S6). Categorization of the genes in our list using DAVID revealed three significantly up regulated biological processes (Benjamini *P*-value <0.05) in the vitamin C treated *wrn-1*(*gk99*) worms compared to untreated wild type worms (Table 5). Major biological processes that were up regulated in vitamin C *wrn-1*(*gk99*) mutant worms included lipid, cellular ketone, and organic acid metabolic processes (Table 5).

**Table 5:** **Biological processes altered in vitamin C treated** ***wrn-1*** **mutant compared to the untreated wild type** ***C. elegans*** **strain**

Up in ***wrn-1***mutant + vitamin C ***vs***untreated N2 ***C. elegans***strain		
Category	Benjamini ***P***-value	Genes
Lipid metabolic process	0.0014	R09B5.6, F35C11.5, F08A8.2, F01D4.2, K11D2.2, F09C8.1, C48B4.1, ZC416.6, Y49E10.18, ZC443.5, T05E7.1, F46G10.4, F01G10.3, C40H1.7
Cellular ketone metabolic process	0.0039	C44E4.3, R09B5.6, ZK829.4, Y73F4A.2, T21F4.1, F08A8.2, R08E5.2, M02D8.4, F01G10.3, C48B4.1, ZC416.6
Organic acid metabolic process	0.0043	C44E4.3, R09B5.6, ZK829.4, Y73F4A.2, T21F4.1, F08A8.2, R08E5.2, M02D8.4, F01G10.3, C48B4.1, ZC416.6

**Figure 3:**
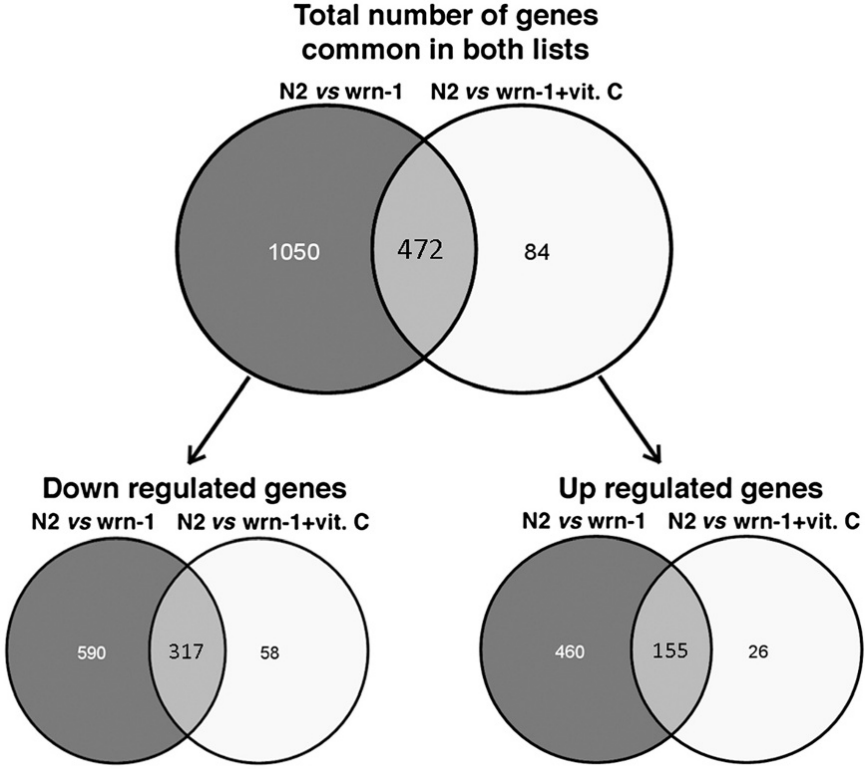
**Venn diagrams showing the number of genes similarly altered in** ***wrn-1*** **mutant** ***C. elegans*** **strains in the presence or absence of vitamin C compared to untreated wild type worms.** The number of genes commonly down regulated or up regulated is also indicated.

We next examined the genes that were commonly altered in untreated and vitamin C treated *wrn-1*(*gk99*) worms taking the global expression of the wild type strain as a reference. The Venn diagrams in Figure 3 indicate on one hand that the expression of 472 genes from the *wrn-1*(*gk99*) worms were changed similarly in the absence or presence of vitamin C when compared to wild type worms. There were 317 and 155 genes down and up regulated, respectively, in the *wrn-1*(*gk99*) worms with or without vitamin C compared to untreated wild type worms. The list of these genes can be found in (Additional file 7: Table S7). DAVID analyses on the commonly up and down regulated genes indicated that the *wrn-1*(*gk99*) mutation increased the expression of genes involved in oxidation-reduction, cellular ketone, organic acid, and lipid metabolic processes (Table 6). These results indicate that vitamin C did not revert the expression of genes involved in these biological processes in the *wrn-1*(*gk99*) worms.

**Table 6:** **Biological processes commonly altered in the** ***wrn-1*** **mutant** ***C. elegans*** **strain in the presence or absence of vitamin C**

Up in ***wrn-1***mutant+/-vitamin C ***vs***untreated N2 ***C. elegans***strain		
Category	Benjamini ***P***-value	Genes
Cellular ketone metabolic process	0.0015	C44E4.3, R09B5.6, ZK829.4, Y73F4A.2, T21F4.1, F08A8.2, R08E5.2, M02D8.4, F01G10.3, C48B4.1, ZC416.6
Organic acid metabolic process	0.0022	C44E4.3, R09B5.6, ZK829.4, Y73F4A.2, T21F4.1, F08A8.2, R08E5.2, M02D8.4, F01G10.3, C48B4.1, ZC416.6
Lipid metabolic process	0.0028	Y49E10.18, R09B5.6, F35C11.5, F08A8.2, K11D2.2, T05E7.1, F46G10.4, F01G10.3, C48B4.1, F09C8.1, C40H1.7, ZC416.6
Oxidation-reduction	0.0391	K08C7.5, R07B7.5, R02D3.1, ZK829.4, K12G11.3, C30G12.2, K09D9.2, T13C5.1, F08A8.2, R04B5.5, C48B4.1

### Potential transcription factors involved in vitamin C response in *wrn-1*(*gk99*) and wild type *C. elegans*

We next performed a transcription factors binding site enrichment analysis on the promoters of the genes altered by vitamin C in both *wrn-1*(*gk99*) and wild type strains. We used publically available ChIP-seq data recently generated for 164 transcription factors in *C. elegans* [27]. Promoter analysis of all the genes differentially expressed by vitamin C in wild type worms revealed a significant enrichment of binding sites for NHR-28 and MAB-5 transcription factors (see the Additional file 8: Table S8). The *C. elegans* NHR-28 protein is an orphan nuclear hormone receptor with zinc finger domains required for DNA binding (http://www.wormbase.org). MAB-5 is a homeodomain transcription factor related to the Antennapedia and Ultrabithorax family of homeodomain proteins. It regulates proliferation, differentiation and morphogenesis in *C. elegans* [28]. The expression of *nhr-28* and *mab-5* genes was not significantly changed in vitamin C treated compared to untreated wild type animals (Additional file 1: Table S1). These results suggest that vitamin C altered the activity of these transcription factors at a post-transcriptional level in wild type animals.

Promoter analysis of all the genes differentially expressed by vitamin C in *wrn-1*(*gk99*) mutant worms revealed a significant enrichment of binding sites for TLP-1, NHR-76, PHA-4 and NHR-28 transcription factors (see the Additional file 9: Table S9). TLP-1 is a zinc finger protein of the Sp family of transcription factor. It is a transcription factor that acts downstream of Wnt signals and that control cell polarity and patterning in *C. elegans* [29]. NHR-76 is a nuclear hormone receptor transcription factor involved in body fat regulation in *C. elegans* [30]. PHA-4 is a transcription factor of the FoxA (forkhead) family that mediates diet-restriction induced longevity in *C. elegans* [31]. It also antagonizes the Target of Rapamycin pathway in *C. elegans* [32]. The expression level of these four transcription factors in *wrn-1*(*gk99*) mutant worms was not significantly changed by the addition of vitamin C (Additional file 1: Table S1). These results suggest that vitamin C altered the activity of these transcription factors at a post-transcriptional level in *wrn-1*(*gk99*) mutant worms.

## Discussion

We have previously observed that homozygous mice lacking exons encoding part of the helicase domain of the Werner syndrome gene product have a reduced mean life span of 16.5% compared to wild type mouse [16]. Importantly, such mutant mice exhibit an abnormally high oxidative stress that can be neutralized by supplementation of vitamin C in drinking water [16]. In fact, vitamin C re-established the normal mean life span of *Wrn* helicase mutant mice. Interestingly, *C. elegans* lacking the *wrn-1* helicase ortholog also have a reduced mean life span compared to wild type worms [19]. The global mRNA expression of whole *wrn-1*(*gk99*) worms by RNA-seq analyses indicated major changes in set of genes involved in overall carbon metabolism (sugars and lipids). These results are consistent with the premature aging phenotype observed in worms with a reduced WRN-1 helicase activity [19, 33]. Although the analysis was performed on mRNA from L4 stage *C. elegans*, altered expression of these genes will have an effect on the duration of adulthood. Indeed, alterations in reactive oxygen species and ATP levels has been observed in adult *wrn-1*(*gk99*) mutant worms [19]. Notably, alteration in the expression of several sets of genes involved in growth and lipid or fatty acid metabolism has not only been observed in cultured *Wrn* mutant mouse embryonic fibroblasts but also in the liver of *Wrn* helicase mutant mice [16, 34]. Importantly, since we noticed a three hours developmental delay in *wrn-1(gk99)* animals compared to wild type, we carefully monitored physiological developmental cues (i.e. vulva and gonad development) in both animal population before harvesting them to perform mRNA analysis (as described in the Methods). Therefore, the change of the expression profile of genes involved in different biochemical pathways observed between *wrn-1*(*gk99*) mutant and wild type animals reflect accurately the difference detected at a specific developmental time point rather than a developmental shift caused by the loss of *wrn-1*. Detailed comparative microscopic and genetic analyses along with global mRNA expression analyses at several developmental stages will be necessary to acquire a better picture of changes as a function of not only chronological but also biological age in *wrn-1*(*gk99*) mutant animals.

Vitamin C treatment increased the mean life span of *wrn-1*(*gk99*) worms to untreated wild type levels. This is similar to the result obtained with mice. Vitamin C supplementation rescued the shorter mean life span of *Wrn* mutant mice [16]. Vitamin C also significantly increased the life span of wild type *C. elegans* (Figure 1). We did not see an increase in the life span of wild type mice supplemented with vitamin C in a previous study [16]. Unlike mice, wild type *C. elegans* do not have the enzyme required for the synthesis of their own vitamin C as far as we can tell. However, *C. elegans* contains three potential cytochromes b561 encoding genes (F55H2.5, F39G3.4 and F39G3.5) [35]. Cytochromes b561 are intrinsic membrane proteins containing two heme molecules, and reducible by ascorbate. They have been suggested to function as electron transporters, shuttling electrons across membranes from ascorbate to an acceptor molecule. The one-electron oxidation product of ascorbate, monodehydro-ascorbate has been shown, at least *in vitro*, to function as an electron acceptor for cytochromes b561. This reaction results in the regeneration of a fully reduced ascorbate molecule [35]. The presence of Cytochromes b561 encoding genes in *C. elegans* suggests that this species could potentially use vitamin C in different chemical reactions [36]. The impact of vitamin C on the levels of different metabolites required for energy or impacting on ROS levels in the body of wild type and *wrn-1*(*gk99*) *C. elegans* is currently under investigation. Interestingly, the expression of the F39G3.4 gene is reduced in *wrn-1*(*gk99*) mutants (Additional file 3: Table S3) but was not changed by the addition of vitamin C (Additional file 4: Table S4). The F39G3.4 gene may be required for the regeneration of ascorbate from monodehydro-ascorbate in *wrn-1*(*gk99*) mutants, a hypothesis that will require confirmation*.*

Our results indicate a major difference in gene expression response to vitamin C between wild type and mutant worms. The list of differentially expressed genes in untreated *wrn-1*(*gk99*) mutants *versus* vitamin C treated *wrn-1*(*gk99*) mutants was very different from the list of genes in vitamin C treated wild type *versus* untreated wild type worms (Additional file 4: Tables S4 and Additional file 5: Table S5). These results indicate that although vitamin C increased the life span of both mutant and wild type worms, it affected different sets of genes in both strains. Another important difference is that the mean life span of vitamin C treated *wrn-1*(*gk99*) worms is shorter (~9 days) than vitamin C treated wild type worms (11 days) (Figure 1). The median life span of *wrn-1*(*gk99*) worms treated with vitamin C was close to untreated wild type worms. The expression profile of vitamin C treated *wrn-1*(*gk99*) worms compared to untreated *wrn-1*(*gk99*) worms revealed down regulation of genes involved in biological processes related to locomotion and anatomical structure development. Upon examination of the global mRNA expression analysis of treated and untreated *wrn-1*(*gk99*) worms (taking the global mRNA expression of wild type as a reference), we found that the expression of 472 genes at the mRNA level was similarly altered in mutant worms compared to wild type worms in the presence or absence of vitamin C. The expression of these genes was affected by the *wrn-1*(*gk99*) mutation but was not reversed by vitamin C suggesting that alteration in expression of these genes is not required for the impact of vitamin C on *C. elegans* life span. These genes are involved in oxidation-reduction, cellular ketone, organic acid, and lipid metabolic processes (Table 6). Thus, vitamin C extended the life span of *wrn-1*(*gk99*) worms by altering biological processes not only involved in locomotion but also in anatomical structure development (Table 4). Such processes are indeed affected at the physiological levels, as a knock down of WRN-1 protein expression by the RNA interference technique results in various developmental defects, including small, dumpy, ruptured, transparent body, growth arrest and bag of worms [33].

The mechanism by which vitamin C alters gene expression in mutant and wild type *C. elegans* is unknown. Our analysis of enrichment for specific transcription factor binding sites in the altered genes in the presence of vitamin C have indicated changes in the activity of hormone receptor-type transcription factors (like NHR-28 and NHR-76) and of PHA-4, a transcription factors which activity depends on pathways affecting the status of several metabolites and/or nutrients in *C. elegans*. One important pathway modulated by metabolites is the CeTOR pathway that affects PHA-4 activity [32]. Our hypothesis is that vitamin C not only affects the redox status of cell in *C. elegans*, but also important metabolites that may be used as ligands for nuclear receptors (lipid derived steroidogenic metabolites) or for signaling molecules that regulate the activity of different transcription factors. Metabolomics analysis with both *wrn-1*(*gk99*) mutant and the N2 wild type strains in the absence or presence of vitamin C is underway and is the scope of another study.

## Conclusions

To conclude, our study indicates that 10 mM vitamin C in the growth media increases the mean life span of the *wrn-1*(*gk99*) mutant and the N2 wild type strains at 25°C. However, the alteration of gene expression by vitamin C is different between *wrn-1*(*gk99*) and wild type strains. Based on our criteria to generate lists of differentially expressed genes between genotypes, we observed alteration in the expression of 1522 genes in *wrn-1*(*gk99*) worms compared to wild type worms. Vitamin C did not significantly modify the alteration of 472 of these genes. Such genes are mainly involved in oxidation-reduction, cellular ketone, organic acid, carbohydrate, and lipid, metabolic processes. Vitamin C, in return, altered the expression of several genes in *wrn-1*(*gk99*) worms involved in locomotion and anatomical structure development. It is interesting to mention that vitamin C can rescue the morphology of liver and adipose tissues of Wrn helicase mutant mice [16]. This report thus provides lists of genes that can potentially affect the healthy aging of not only wild type but also *wrn-1*(*gk99*) worms upon genetic manipulation in the presence of vitamin C. It also provides important clues on the biological processes that vitamin C affects to increase life span in *C. elegans*.

## Methods

### *Caenorhabditis elegans*strains

All *C. elegans* strains were maintained as described [37]. The *wrn-1(gk99)* strain was obtained from the *C. elegans* Genetics Center (University of Minnesota, St Paul, MN) and was out-crossed four times with the wild type N2 strain to remove possible unrelated mutations. The *wrn-1(gk99)* contains a 196 bps deletion that inhibits the expression of the protein [38]. The primers used to genotype this strain are 5′-CTGGCTGTAACTGCACCTGA-3′ and 5′-AAATGGGAGGGAAAGAGCAT-3′. To measure life span, worms were transferred to fresh plates and were grown at 25°C. Death was scored by absence of any movement after several light pokes with a platinum wire.

### Extraction of total RNA and sequencing

Eggs were isolated by standard bleach treatment and hatched overnight at room temperature. The resulting L1 larvae were then distributed over NGM plates supplemented or not with 10 mM vitamin C (Sodium L-ascorbate) (Sigma-Aldrich, ST-Louis, MO) and grown at 25°C. A concentration of 10 mM ascorbate was chosen in this study as it has previously been shown that such a concentration did not influence the survival of *C. elegans* [25]. The developmental stage of the animals was monitored visually until the worms reached the larvae L4 stage. Animals were precisely staged by observing vulval and gonad formation by Nomarski optics before collecting the animals for analysis as described previously [39]. Worms were spun down in an eppendorf tube and lysed in TRIZOL (Invitrogen, Carlsbad, CA) to extract total RNA. RNA quality was checked with an Agilent Bioanalyzer 2100 and libraries were made using the Illumina’s TruSeq v2 kit according to the manufacturer’s recommendations. The RIN (RNA Integrity Number) of all samples was above 8.0. Fifty base pairs paired end sequencing was performed on the HiSeq 2000 machine form Illumina.

### RNA-seq analyses

The data discussed in this publication have been deposited in NCBI’s Gene Expression Omnibus http://www.ncbi.nlm.nih.gov/geo/ [40] and are accessible through GEO Series accession number GSE54173. Table 1 contains the read statistics for the raw reads coming off the sequencer. Detailed statistics on the quality of the reads were calculated with FastQC (http://www.bioinformatics.babraham.ac.uk/projects/fastqc/). The 50 base pairs raw sequences were aligned on the *C. elegans* ce10/W220 genome with TopHat [41] using the Ensembl annotations provided with the Illumina iGenomes. The htseq-count software (http://www-huber.embl.de/users/anders/HTSeq) was used to count the number of reads aligned to each gene. These counts were then normalized relative to the sequencing depth with DESeq [42]. DESeq was also used to calculate the fold changes, log fold changes, and significance of the changes for each comparison.

Enrichments for specific biological functions using the DAVID (Database for Annotation, Visualization, and Integrated Discovery) web site [43] were considered significant with a *P*-value smaller than 0.05.

### Validation of mRNA expression

The quantitative measure of selected mRNA expressions was performed by qRT-PCR. Worms were spun down in an eppendorf tube and lysed in TRIZOL (Invitrogen, Carlsbad, CA) to extract total RNA. PolyA + RNA was isolated with the RNeasy plus mini kit (Qiagen, Mississauga, ON). The cDNAs were obtained by reverse transcription using the specific 3′-primer on total RNA (Additional file 2: Table S2 for primer design). cDNA were then subjected to qPCR reaction using PerfeCTa® SYBR® Green FastMix®, Low ROX™ (Quanta Bioscience, Gaithersburg, MD) according to manufacturer instruction. At the end of each run, samples were analyzed on a 2% agarose gel to determine the quality of the amplification. Primers used for qRT-PCR are listed in (Additional file 2: Table S2).

### modENCODE transcription factors binding site enrichment analysis

We downloaded a list of 164 significant ChIP-seq peaks mapped on the ws220 *C. elegans* genome from the modENCODE FTP website (ftp://data.modencode.org/) [27]. Using this significant list of peaks, we first defined for every transcription factors a list of genes located within 500 bp of at least one significant peak. Using this list of genes we computed the overlap enrichment scores between the list of genes targeted by the transcriptions factors and the list of significantly differentially expressed genes identified in our *wrn-1* and wild type gene expression experiments for up-regulated, down-regulated and all significant genes. The enrichment was evaluated based on a one-sided hypergeometric distribution. We consider an enrichment between genes targeted by a transcription factor and the list of genes modulated by vitamin C to be significant if the Benjamini-Hochberg adjusted p-value is lower than 0.05.

## Electronic supplementary material

Additional file 1: Table S1: Normalized counts for each biological replicate. This list gives the normalized number of reads for each gene in each replicate. (XLS 5 MB)

Additional file 2: Table S2: Primers used for the quantitative RT-PCR. (XLS 18 KB)

Additional file 3: Table S3: List of genes found to be differentially expressed in the *wrn-1* mutant compared to the wild type N2 worms. The columns labeled baseMeanA and baseMeanB represent the mean number of reads (from the normalized counts of Additional file 1: Table S1) for the N2 wild type and the *wrn-1(gk99)* strains, respectively. (XLS 318 KB)

Additional file 4: Table S4: List of genes found to be differentially expressed in the vitamin C treated *wrn-1* mutant compared to untreated *wrn-1* mutant worms. The columns labeled baseMeanA and baseMeanB represent the mean number of reads (from the normalized counts of Additional file 1: Table S1) for the untreated *wrn-1(gk99)* and the vitamin C treated *wrn-1(gk99)* worms, respectively. (XLS 79 KB)

Additional file 5: Table S5: List of genes found to be differentially expressed in the vitamin C treated wild type N2 compared to untreated wild type N2 worms. The columns labeled baseMeanA and baseMeanB represent the mean number of reads (from the normalized counts of Additional file 1: Table S1) for the untreated N2 wild type and the vitamin C treated N2 wild type worms, respectively. (XLS 77 KB)

Additional file 6: Table S6: List of genes found to be differentially expressed in the vitamin C treated wrn-1 mutant compared to untreated wild type worms. The columns labeled baseMeanA and baseMeanB represent the mean number of reads (from the normalized counts of Additional file 1: Table S1) for the untreated N2 wild type and the vitamin C treated *wrn-1(gk99)* worms, respectively. (XLS 128 KB)

Additional file 7: Table S7: Genes differentially expressed in a similar way in *wrn-1* mutant worms incubated with or without vitamin C compared to the untreated wild type N2 worms. (XLS 64 KB)

Additional file 8: Table S8: List of transcription factors potentially binding to the promoters of genes altered by vitamin C in wild type worms. (XLS 42 KB)

Additional file 9: Table S9: List of transcription factors potentially binding to the promoter of genes altered by vitamin C in *wrn-1* mutant worms. (XLS 36 KB)
